# Acknowledgement to Reviewers of *Microorganisms* in 2017

**DOI:** 10.3390/microorganisms6010007

**Published:** 2018-01-12

**Authors:** Microorganisms Editorial Office

**Affiliations:** MDPI AG, St. Alban-Anlage 66, 4052 Basel, Switzerland

Peer review is an essential part in the publication process, ensuring that *Microorganisms* maintains high quality standards for its published papers. In 2017, a total of 77 papers were published in the journal. Thanks to the cooperation of our reviewers, the median time to first decision was 18 days and the median time to publication was 45 days. The editors would like to express their sincere gratitude to the following reviewers for their time and dedication in 2017:
Addington, CarolineKirst, HenningAfonso, Clélia N.Klewicka, ElzbietaAlber, Birgit E.Klose, HolgerAlexeyev, OlegKolodkin-Gal, IlanaAlspaugh, J. AndrewKuberski, TimÁlvarez, María S.Kubicek, ChristianAntunes, AndréKuuskeri, JaanaArgyri, Anthoula A.LaMontagne, MichaelArroyo-Lopez, Francisco NoeLaPointe, GisèleAsensio, MiguelLauder, BobAvrani, SaritLauerma, AnttiAxel, ClaudiaLauzon, Carol R.Axmann, Ilka MariaLea-Smith, DavidAymerich, TeresaLefticariu, LilianaBaldwin, SusanLev, SophieBarral Silva, MariaLewis, Jeffrey A.Barriuso, JorgeLlanes, CatherineBassi, DanielaLuna, Gian M.Baumgartner, KendraLyons, Jennifer L.Bemer, PascaleLytton, TimothyBenedetti, AnnaMaciá-Vicente, Jose G.Benito, SantiagoMaki, JamesBevilacqua, AntonioMcBain, AndrewBignell, DavidMcDowell, AndrewBird, AmandaMenendez, EstherBizukojc, MarcinMercer, JasonBlacutt, AlexMoreira, CristianaBleve, GianlucaMoriarty, Thomas FintanBoll, JosephMorley, KristaBomberg, MalinMuzzalupo, InnocenzoBonfield, Tracey L.Nagata, YujiBongiorni, LuciaNakajima, MasamiBorges, FrédéricNemerow, GlenBorghese, RobertoNishiyama, KeitaBrand, LarryNobre, TaniaBrown, AmandaOelschlägel, MichelCasotti, RaffaellaOlesen, KjeldCassier-Chauvat, CorinneOlson, Michael E.Céline, LarocheÖstman, ÖrganCenci-Goga, Beniamino T.Panagiotopoulos, Ioannis A.Chai, YunrongPatrauchan, Marianna A.Ciofu, OanaPérez-Tanoira, RamónCocconcelli, Pier SandroPetrova, Olga E.Cole, JeffPlacido, JerssonCollett, AndrewPruess, Birgit M.Congestri, RobertaPucciarelli, SandraConti, HeatherQuirós, Luis M.Cordero, Radamés J.B.Radek, RenateCuesta, IsabelRamakrishnan, SrinivasanDe Curtis, FilippoRanadheera, C. SenakaDe La Fuente-Núñez, CésarRemize, FabienneDelbarre-Ladrat, ChristineRichard, PeterDhami, Navdeep KaurRivilla, RafaelDien, BruceRodriguez, FranciscoDomingues, SaraRoselli, MariannaDonovan, SharonSaleem, MuhammadDrosinos, EleftheriosSalmerón, IvanDuh, Pin-DerSanches, SandraDunfield, PeterSánchez Mainar, MaríaDurazzo, AlessandraSanford, RobertEhrmann, Matthias A.Satora, PawelEl Khalloufi, FatimaSchaberle, Till F.Elisashvili, VladimirSchröder, CarolaEne, Iuliana VeronicaSerrano, AurelioEngelen, AschwinShafer, William M.Enjalbert, FrancisShalaby, SamerEvilia, CarynShemesh, MosheFarnoud, AmirShoenfeld, YehudaFarrell, HazelShumway, SandyFetissov, Sergueï O.Siddiqui, KhawarFilannino, PasqualeSilva, Gabriela JorgeFinley, Rita L.Sinha, RohitaFiocco, DanielaSipponen, Mika HenrikkiFixen, KathrynSkraban, JureForsyth, Christopher B.Sommer, StephanFothergill, JoanneSpano, GiuseppeFraga, IreneSpillmann, DorotheGalanis, AlexStella, SimoneGile, GillianStępień, KrzysztofGiron, Jorge AlbertoStrangman, WendyGonzalez, AridaneStrobel, Gary A.Gorrell, RebeccaSuda, ShoichiroGrbin, PaulTan, Kai SooGrosse, StephanTaylor, JoeGustafson, JohnTerzi, ValeriaHallsworth, John E.Thompson, AndrewHanson, BuckTirloni, EricaHare, KimTitz, AlexanderHauge, Sigrun J.Topakas, EvangelosHebert, E.M.Torriani, SandraHemp, JamesTripathi, AshootoshHennequin, ClaireTseng, Chun-ChiehHill, DarrylTuovinen, OlliHillmann, FalkUppuluri, PyriaHolmes, Dawn EVan Bambeke, FrançoiseHorie, MasanoriVan Laar, TriciaHosório, HugoVan, Thi Thu HaoHu, HonghuaVaneechoutte, MarioHu, QingVarela, MartaIanieri, AdrianaVerma, MadanIbrahim, FandiVon Bergen, MartinInsam, HeribertWan, XuehuaIorizzo, MassimoWasmund, KennethJackson, AndrewWeighardt, HeikeJackson, Stephen A.Wemheuer, FranziskaJanbon, GuilhemWhite, JamesJass, JanaWojcieszyńska, DanutaJehmlich, NicoWolf-Rainer, AbrahamJennings, JessicaWozniak, KarenJohnston, SimonXimenes, EduardoJorand, Frédéric P.A.Yan, AixinJordao, LuisaYokawa, KenKaminskyj, Susan G.W.Zan, JindongKenyon, WillianZonaro, Emanuele

